# The geosimulation of West Nile virus propagation: a multi-agent and climate sensitive tool for risk management in public health

**DOI:** 10.1186/1476-072X-7-35

**Published:** 2008-07-07

**Authors:** Mondher Bouden, Bernard Moulin, Pierre Gosselin

**Affiliations:** 1 Department of Computer Sciences and Software Engineering, Laval University, Quebec, G1V 0A6, Canada; 2 Centre for Research in Geomatics, Laval University, Quebec, G1V 0A6, Canada; 3 Institut national de santé publique du Québec (INSPQ), 945 avenue Wolfe, Quebec, G1V 5B3, Canada; 4 Centre hospitalier universitaire de Québec (CHUQ), 2705, boulevard Laurier, Quebec, G1V 4G2, Canada

## Abstract

**Background:**

Since 1999, the expansion of the West Nile virus (WNV) epizooty has led public health authorities to build and operate surveillance systems in North America. These systems are very useful to collect data, but cannot be used to forecast the probable spread of the virus in coming years. Such forecasts, if proven reliable, would permit preventive measures to be put into place at the appropriate level of expected risk and at the appropriate time. It is within this context that the Multi-Agent GeoSimulation approach has been selected to develop a system that simulates the interactions of populations of mosquitoes and birds over space and time in relation to the spread and transmission of WNV. This simulation takes place in a virtual mapping environment representing a large administrative territory (e.g. province, state) and carried out under various climate scenarios in order to simulate the effects of vector control measures such as larviciding at scales of 1/20 000 or smaller.

**Results:**

After setting some hypotheses, a conceptual model and system architecture were developed to describe the population dynamics and interactions of mosquitoes (genus *Culex*) and American crows, which were chosen as the main actors in the simulation. Based on a mathematical compartment model used to simulate the population dynamics, an operational prototype was developed for the Southern part of Quebec (Canada). The system allows users to modify the parameters of the model, to select various climate and larviciding scenarios, to visualize on a digital map the progression (on a weekly or daily basis) of the infection in and around the crows' roosts and to generate graphs showing the evolution of the populations. The basic units for visualisation are municipalities.

**Conclusion:**

In all likelihood this system might be used to support short term decision-making related to WNV vector control measures, including the use of larvicides, according to climatic scenarios. Once fully calibrated in several real-life contexts, this promising approach opens the door to the study and management of other zoonotic diseases such as Lyme disease.

## Background

The WNV is a flavivirus which was isolated for the first time in 1937. Its name comes from the district of West Nile in Uganda. It was detected in human, birds and mosquitoes in Egypt at the beginning of the fifties, and has then been found in various European countries [1]. It is however only with the important 1996 human epidemic in Bucharest, Romania, that WNV became a concern for public health. Moreover, there is no specific treatment of the disease and no vaccine is yet available for humans. The WNV was detected on the American continent in 1999 and more specifically in New York [2]. In Canada, WNV reached southern Ontario in 2001, while the first human cases were detected in August 2002 [3].

WNV made its appearance in Quebec in July 2002. The virus was then propagated, like everywhere else, by the intermediary of mosquitoes and birds. The expansion of this epizooty forced the Government of Quebec to adopt an intervention plan which included in 2003 the implementation of a multi-faceted surveillance system [4]. This system brought together field data on human, avian and entomological infection and deaths.

While these monitoring activities were undertaken to better understand the epidemiology of WNV and the level of risk it can represent for the human population, they do not allow for forecasts of the probable propagation of the virus on the territory. Such a forecast, if it proved to be reliable, would allow public health authorities to initiate preventative actions at the right time and places and at the appropriate level of expected risk. Currently, one main control activity is larvicide spraying in urban and rural settings in order to reduce the population of mosquitoes infected with WNV. However, it remains difficult to determine the at-risk zones on a scientific basis and the efficacy of such measures has been challenged [5], not to mention their high cost and environmental impacts. The identification of vulnerable zones and risk levels in due time remains a significant challenge for public health management due to the complexity of the phenomena related to the virus transmission.

Multi-agent geosimulation is an artificial intelligence modeling approach which might be used to develop public health management tools in order to anticipate the progression of the disease and to assess various intervention scenarios. This approach makes it possible to simulate the behaviours of thousands of agents in geo-referenced virtual spaces. The MAGS System (Multi-Agent GeoSimulation) recently developed by Dr. Moulin's *Groupe de Recherche en Informatique Cognitive *at Laval University, can be used to create such simulations in virtual environments generated with georeferenced data obtained from geographic information systems (GIS). These agents are characterized by cognitive capacities such as perception of the environment and its content, autonomous navigation and reasoning [6]. Although one of the first applications of MAGS was related to the simulation of crowd behaviours in urban environments, MAGS is a generic platform allowing the simulation of several types of behaviours in various geo-referenced virtual environments. For example, it has already been used to simulate the behaviour of consumers visiting shopping centers and firemen intervention plans to contain the propagation of forest fires [7].

The main objective of the WNV-MAGS Project reported in this paper, was to develop a system to simulate the behaviours and interactions of populations of indicator birds and of mosquitoes involved in the propagation and transmission of the WNV, taking into account the characteristics of the geographic environment. This simulation takes place in a virtual cartographic world representing a large territory (southern part of the province of Quebec, Canada). The simulation also takes into account various climatic scenarios and regimens of larvicide treatments.

In Section 2, we present an overview of the phenomena which are linked to the spread of WNV. Then, we present the conceptual model which was developed after setting some carefully chosen hypotheses. Next, we present the geosimulation of the populations of interest, using agents' roosts to represent the dynamics of the bird populations and an intelligent density map to represent the populations of mosquitoes. Some short-term climate scenarios and the calibration of the system are also presented in this section. In Section 3, we present a conclusion and some new work currently underway. In Section 4 we briefly present the design method used to develop the system, including the conceptual architecture and an overview of the mathematical model formalizing the evolution of relevant populations. We also comment upon the quality and availability of data used to feed the system. Finally, we briefly present the implementation context of the system.

## Results and discussion

### Overview

**Figure 1:**
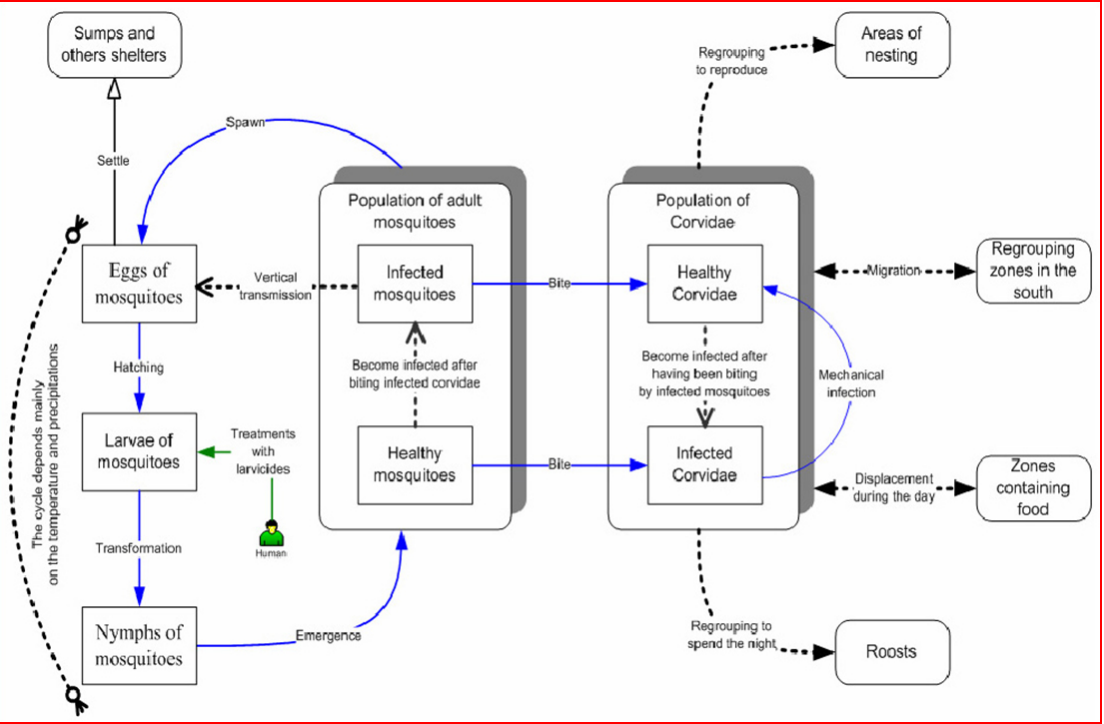
Overview of phenomena of interest for the WNV-MAGS Project.

Figure 1 presents a synthetic view of the phenomena which are involved in the spread of WNV, as adapted for the Quebec context [8]. Indeed, there are mainly two populations involved in the transmission of the WNV: the population of mosquitoes (*Culex sp*.) and the population of birds. In this paper, we mainly consider the *Corvidae *family and more specifically crows which have been chosen by public health authorities as indicator birds for the WNV. Mosquitoes spawn eggs in sumps and other shelters. The larvae hatch from eggs and evolve into nymphs that emerge to become adult mosquitoes. This cycle mainly depends on temperature and humidity [9]. Besides, human intervention can reduce the population of mosquitoes through larvicide treatments (e.g. Methoprene) in order to kill larvae. The transmission of the WNV occurs mainly mosquitoes biting birds. An infected mosquito can infect a bird, which can in turn infect healthy mosquitoes that will subsequently bite the infected bird before its death [10].

Regarding the populations of *Corvidae*, their spatial and temporal characteristics depend on geographic areas and on the periodicity of displacements and grouping. During early spring bird couples spread over the whole territory and remain for few months around their nesting areas. By the end of July, which happens to be the very beginning of human infections in Quebec [3], *Corvidae *change their social behaviour and regroup in roosts at night. During the day, the birds fly to surrounding areas in search of food, but they go back to the roosts at night [11]. At the end of fall, many of them migrate to warmer areas south of the province [12]. Furthermore, the transmission of WNV to the populations of *Corvidae *can occur either by mechanical infection (an infection after a direct contact between birds) or through the biting of a healthy *Corvidae *by an infected mosquito (Figure 1).

Since we wanted to simulate the progression of the WNV infection involving a large number of individuals of two main species interacting in a particular geographic region, we selected a geosimulation approach which allows for the study of the spatial and temporal characteristics of the populations' interactions in a virtual geographical environment [6]. However, given the enormous complexity involved in representing such phenomena and the lack of detailed data, we had to raise a number of reasonable simplifying hypotheses with regard to the species of mosquitoes and Corvidae of interest, to the factors influencing the evolution of these populations, the geographical region selected for the analysis, the period of simulation and the space-time scale. These hypotheses led us to identify a set of key parameters to carry out the simulations, based on the epidemiologic and surveillance experience with WNV in North America and more precisely in the province of Quebec [4]. For example, considering the availability of surveillance data, we selected the American crow as the main indicator bird species; and the *Culex pipiens *and *Culex restuans *as the main mosquito's species susceptible to bite crows (and possibly humans). Another example is the period of simulation for the WNV propagation: July 1st to October 1st was selected as the critical time window during which human cases have appeared in Quebec so far (Table 1).

**Table 1:** Specifications of the simulation parameters.

**Parameters**		**Specifications**
Considered species		Representative species of *Corvidae *populations: *Corvus brachyrynchos *(American crow). Other birds are also considered. Representative species of mosquito's populations: *Culex pipiens *and *Culex restuans*.
Main factors influencing the behaviours	*Culex*	Climatic conditions (primarily the temperature and precipitations).
	crows	Zones and periodicity of displacements and grouping.
Geographical region		The Ecumene zone of the following administrative areas: Québec, Chaudière-Appalaches, Mauricie-Centre du Québec, Montérégie, Estrie, Montréal-centre, Laval, Laurentides, Lanaudière et Outaouais.
Period of simulation		We decided to simulate the WNV propagation from July 1 until October 1.
Temporal scale		An interval of a daily step with a weekly assessment.
Space scale		The micro-space scale specifies the size of a pixel (4 km^2^). The macro-space scale give an idea on the spread of the WNV propagation in order to bring a help to the decision-making concerning the larvicides treatments (interpolated by aggregation of pixels between 1 and 50 km^2^).

### Conceptual model

**Figure 2:**
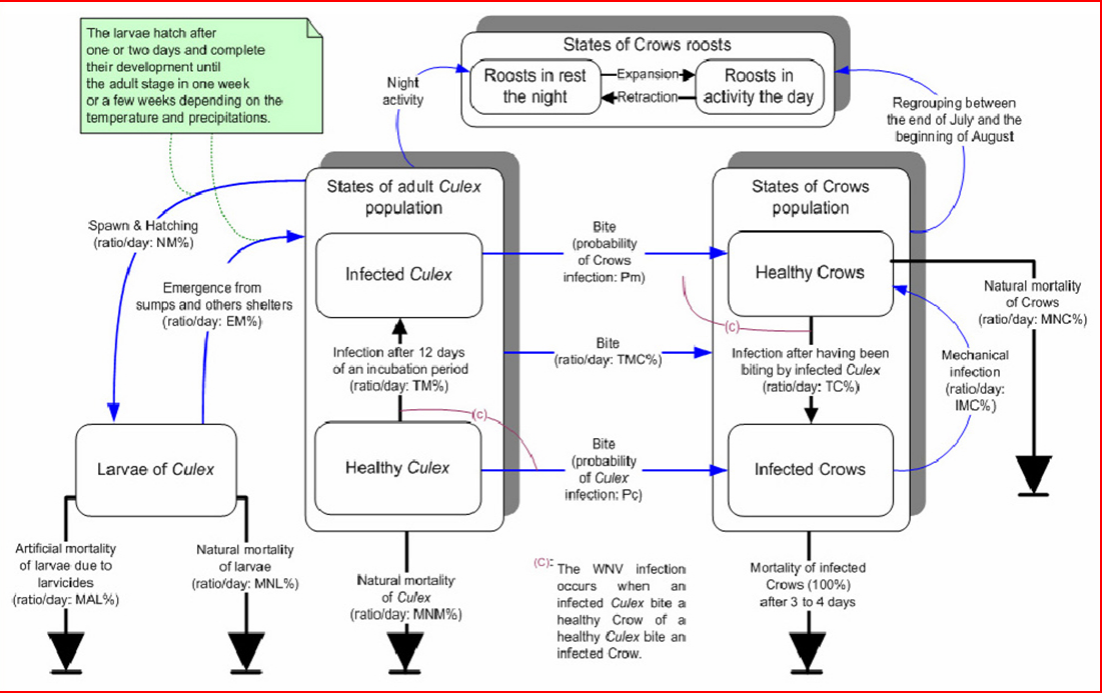
Conceptual model representing the dynamics of *Culex *and Crows.

The objective of the conceptual model is to introduce a synthetic view of the phenomena of interest while taking into account the above mentioned simplifying hypotheses (Figure 2). Let us briefly comment upon this model which represents the evolution and interactions of *Culex sp*. (*pipiens*/*restuans*) and crows. Moreover, we simplified the biological cycle of *Culex *to only consider the change from a larval state to an adult one. From a public health management's point of view, these two states are the most important ones since the virus is spread by adult females and treatments against the progression of the WNV are carried out using larvicides. This simplification has been validated by domain experts (see below). In addition, considering the spatial dynamics of crow populations, we selected the period of the year when *Corvidae *regroup in roosts. In our model, a roost is considered as the spatial extension of an aggregate of crows (a sub-population of crows which gathers in this roost for the period of the year of interest). During the day, crows fly a variable distance from the roost in search of food, and return at night. Hence, the spatial phenomenon of gathering and dispersion of this sub-population of crows can be represented in a synthetic way in the form of an expansion and a contraction of the area occupied by this sub-population. The surface over which the birds spread during the day ("roost expansion") depends on the roost size. Consequently, we can take into account the variable density of crows in this dynamically changing area. Another fact to consider is that the *Culex *mosquito has a mostly nocturnal activity [13]. Therefore, the crows located in roosts at night will be good targets for them. Moreover, preset variables have been used in order to compute parameters such as the infection probabilities and the mortality proportions. This conceptual model has been validated by domain experts (from the *GDG *Company, *Université de Sherbrooke *and *Université du Québec à Trois Rivières *– UQTR) and was used to orient the development of the geosimulation tool.

### Geosimulation of the populations

According to our conceptual model, the progression of the WNV infection involves a large number of individuals of two main species and their interactions depend on the probabilities of finding sub-populations of these species within the same geographic areas at specific times. We already mentioned the interest of using a multi-agent geosimulation approach in such a context. However, we had to adapt it to take into account the large geographic area of interest and the very large size of the involved populations, especially for *Culex*.

**Figure 3:**
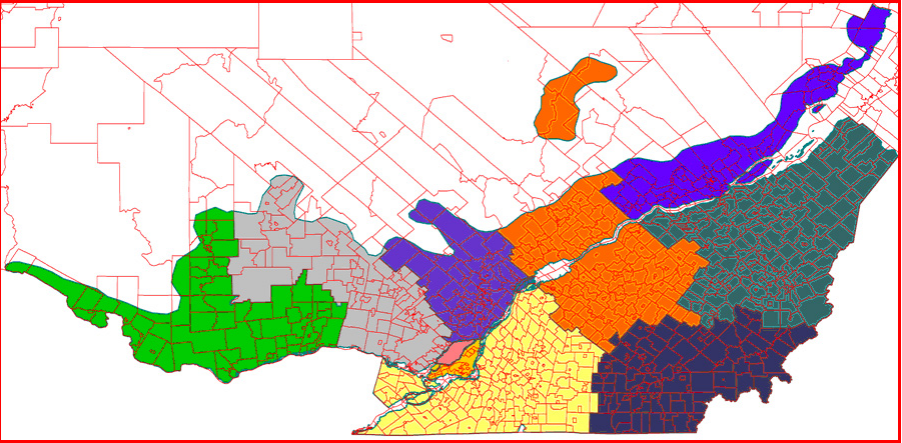
The geographic area of interest which contains all the municipalities belonging to the ecumene (Southern Quebec, Canada).

To create the virtual geographic environment representing the studied region (Figure 3), we first collected geo-referenced data and generated the various spatial data layers needed by the MAGS platform. Then, we modelled the two populations involved in the transmission of the virus as well as their locations in this virtual environment. Indeed, the population of *Culex *represents an extremely large number of individuals and cannot be represented using individual agents. Instead, we decided to model the mosquito population as an 'intelligent density map' which is characterized by population data being attached to reference areas (municipalities) in the virtual space. The idea is to associate to each reference area a list of variables corresponding to the numbers of the different categories of mosquitoes (larvae, healthy and infected adults) located in this place. These numbers evolve during the simulation as a consequence of various parameter changes (temperature, degree of humidity resulting from rainfall, etc.) as well as encounters with crows. For the population of crows, we used agents to model groups of crows associated with specific areas where roosts have been observed in the field. The interactions of the two populations have also been modeled thanks to the geosimulation which enables the system to automatically determine the places and times where groups of crows (pertaining to roosts) will cross areas in which the *Culex *sub-populations are located.

#### Intelligent density map

**Figure 4:**
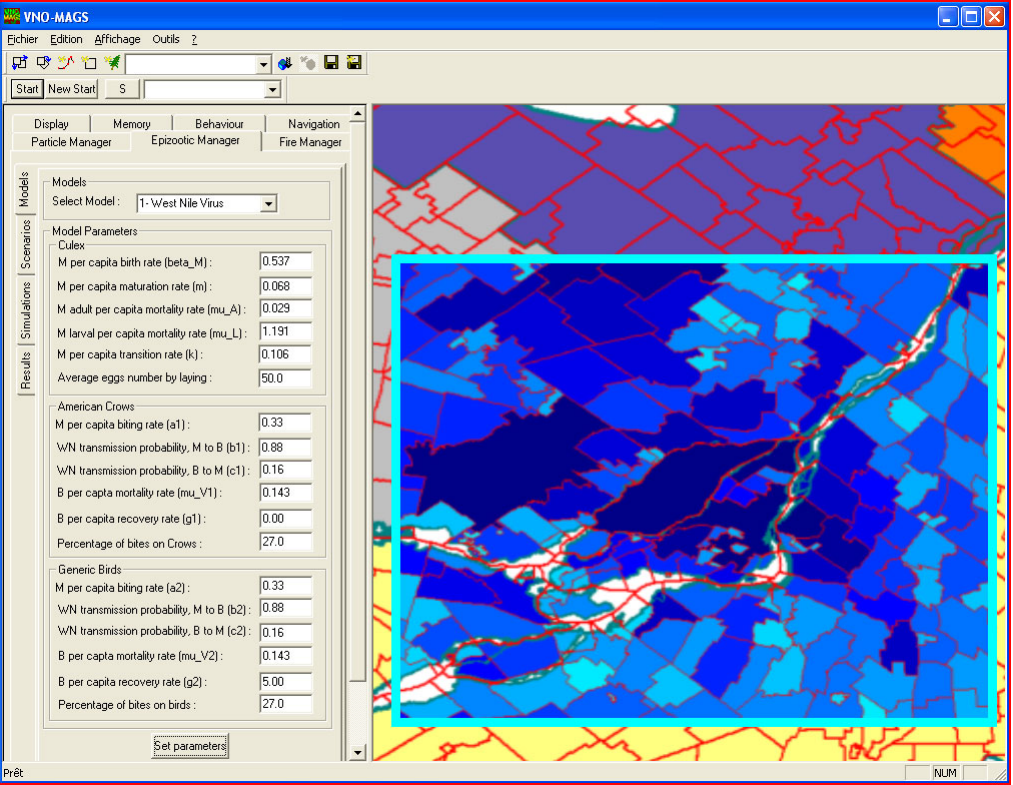
Using an intelligent density map to represent the population of *Culex*.

The populations of *Culex *do not move much and they are present practically everywhere in the selected territory [3,14]. Because of the extremely large number of individual mosquitoes, we represent sub-populations of mosquitoes as characteristics of the virtual geographic environment and we use what we call an "intelligent density map" that represents the distribution of *Culex *sub-populations over the different reference areas depending on the geographic characteristics and the locations of favourable habitats for mosquitoes. This intelligent density map is a kind of cellular automaton associated with rules that enable the system to simulate the evolution of the different categories of mosquitoes (larvae, adults, healthy, infected, etc.) in each reference area under the conditions that influence the mosquitoes' life cycle (temperature and precipitations). The system gets these conditions either from actual meteorological data (from specific databases: see Section 4.3) or from the parameters set in scenarios that the user wants to explore. This map contains the polygons representing all the municipalities of the Quebec province (the reference areas). On the user's screen, the color of each municipality changes according to the relative densities of the *Culex *populations (ratios of healthy, and infected adults) computed by the simulator (Figure 4).

#### Agents' roosts

A roost synthesizes the behaviour of a group of crows. It is modeled by an agent having some initial characteristics such as the number of individuals, the position of the roost on the map, and the maximum radius of its expansion area. These characteristics are computed using various field data as presented in Section 4.3. Moreover, this agent inherits from all the functionalities of MAGS agents. For example, it uses some behaviour rules in order to model how crows scatter around the roost. In addition, an *operating range parameter *is computed for each roost in order to estimate the maximum distances covered by the crows when they search for food during the day.

**Figure 5:**
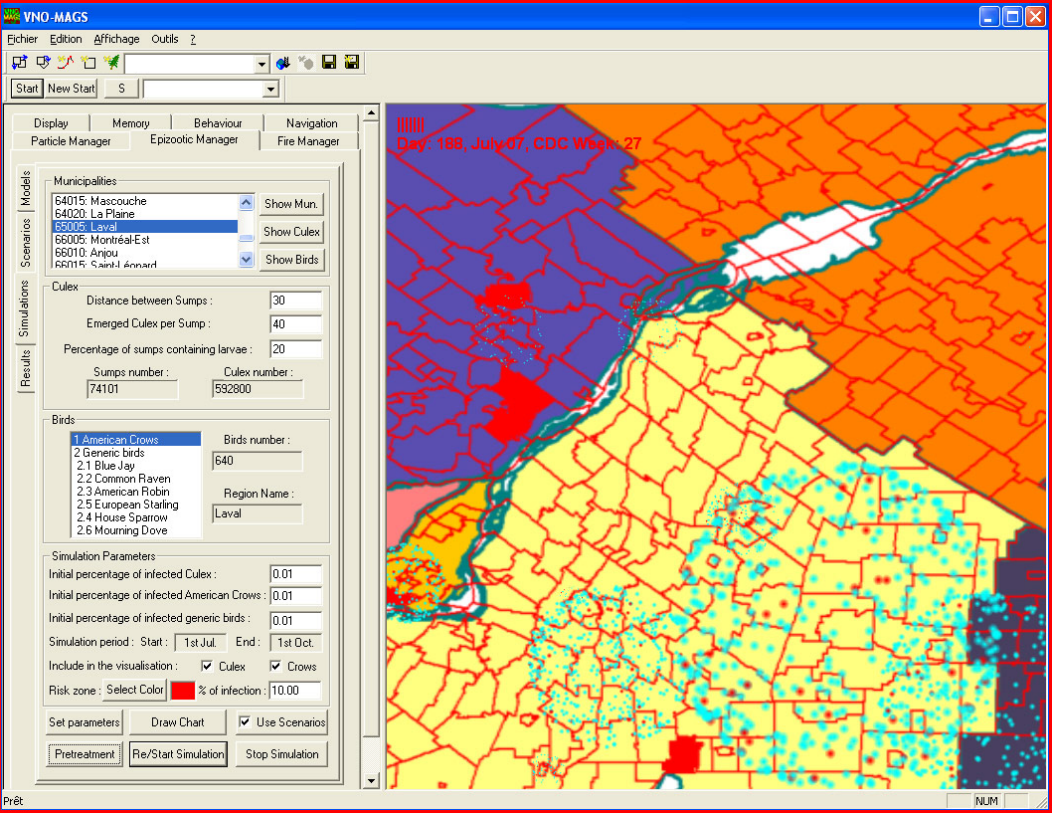
Using roosts to represent the populations of crows.

Each roost agent is implemented as a particle system [15] which simulates the way crows spread around a roost during the day. Hence, such particle systems behave as agents, as described above. Each particle represents one or several crows, depending on the number of individuals attached to the roost. In Figure 5 we can see a snapshot of a simulation in which roosts are displayed as "clouds of blue particles". Each particle has different characteristics (velocity, direction of movement) that enable it to travel at a distance from the roost location during a number of simulation steps representing a day. Hence, the set of particles associated with a given roost covers a circular area with a maximal radius set by the *operating range parameter*. We calibrated the parameters of the particle system by computing the density of crows in the area covered by the expansion of the roost and comparing it to observed field data and other estimated data.

#### Interaction between the two main populations

The interaction between the two main populations is a very important functionality of our system, since it is the way of representing the evolution of the infection. Indeed, while traveling in the geographic space, one or several crows represented by a particle can cross areas in which *Culex *mosquitoes are located. Consequently, there is a probability that some of these crows will be bitten. Technically, in order to determine the probabilities of encounters between mosquitoes and crows, the corresponding particle takes into account the characteristics of the *Culex *population associated with each reference area of the 'intelligent density map' over which it travels. Therefore, the system can estimate the number of infected individuals, based on the likelihood that a number of individual crows be bitten by mosquitoes and be infected with WNV (using the equations of the mathematical model described in Section 4.2). Moreover, the user can visualize the extent of the spread of WNV on the map in different ways. The system can either change the color of the particles representing the infected crows or the color of the polygon representing a municipality containing a high density of infected *Culex*.

#### The influence of other bird species

Our initial simulations involved the two main species of American crows and *Culex pipiens*/*restuans *that we selected and that enabled us to apply Wonham's mathematical model [16] (see Figure 12a in section 4.2). We quickly found out some limits with this model, since it does not take into account the influence of temperature on the evolution of mosquito's populations. When we applied this model, it led, after a number of iterations, to the complete "extinction of crows". This, obviously, does not conform to reality, although a dramatic decrease of *Corvidae *populations have been observed in recent years due to the spread of WNV [17].

Hence, we proposed an extension of this model which enables us to model several species of birds and to take into account the impact of the temperature in terms of cumulated degree-days which influence some parameters of the model (see Figure 12b in section 4.2). We cannot discuss here the details of such a model and its implementation (for more details, see [18]). In the current experiments, we modeled the interactions between crows and mosquitoes as described in the previous section. Since surveillance systems provide data about crows as indicator birds, we used this species to set the simulation parameters and to calibrate the system. However, we added other bird species in the simulation to increase "the biting opportunities" for mosquitoes, so that the "crow population" does not become extinct by the end of the simulation period. Indeed, this is a plausible hypothesis: mosquitoes bite other birds as well as crows.

We thus introduced in the WNV-MAGS system another 'global' population of birds, that we called "generic birds" (Common Raven, Blue Jay, American Robin, House Sparrow, European starling and Mourning Dove) which are resident in the municipalities and known to carry WNV [17]. This population of generic birds appears in the mathematical model with similar equations as those used for the crows (each bird population is represented by a different index *j *in the equations: see Figure 12b in section 4.2). However, the parameters for each bird family may be different. Due to lack of data, we currently set some average parameters to the equations of the "generic birds". Getting more accurate parameters will require further research from bird specialists. In the simulation, one distinction that we established between crows and "generic birds" is that we assumed that birds do not move outside the municipality (as crows may do while flying away from the roosts). Hence, generic birds stay in contact with the same mosquito population during the simulation. Indeed, this is a simplification. Since our system is parameterised, we will be able to introduce parameters for other bird species as soon more precise data will be available with respect to the ecology and epidemiology of other birds affected by WNV.

#### Using various short-term climate scenarios

**Figure 6:**
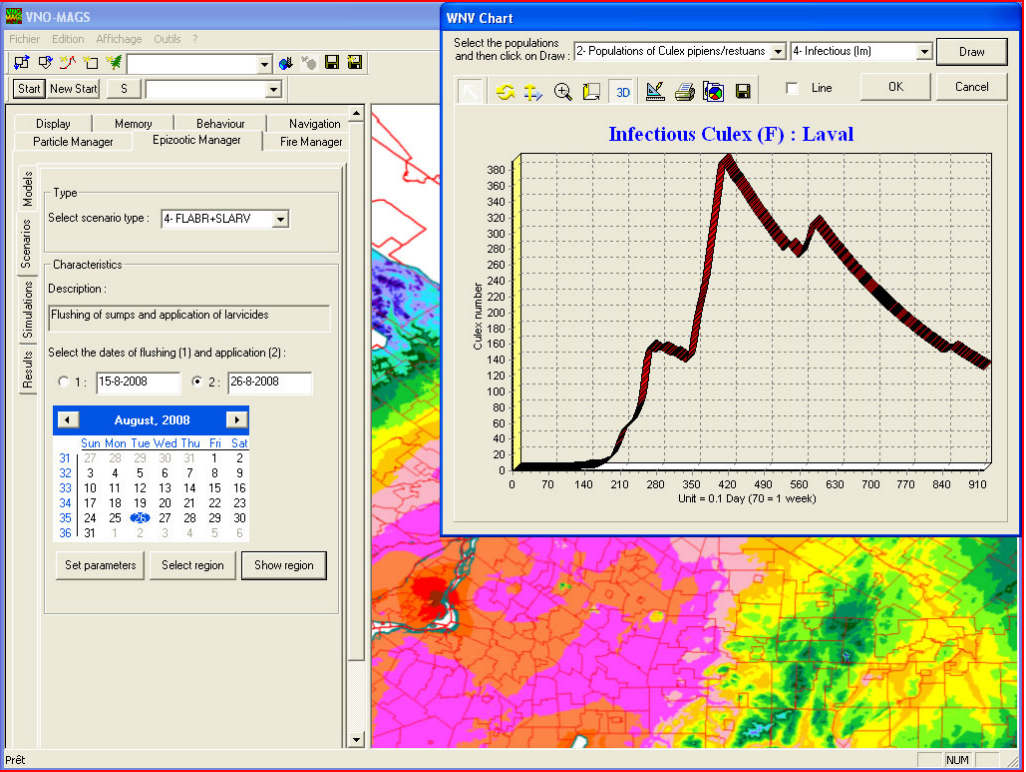
Using management scenarios.

**Figure 7:**
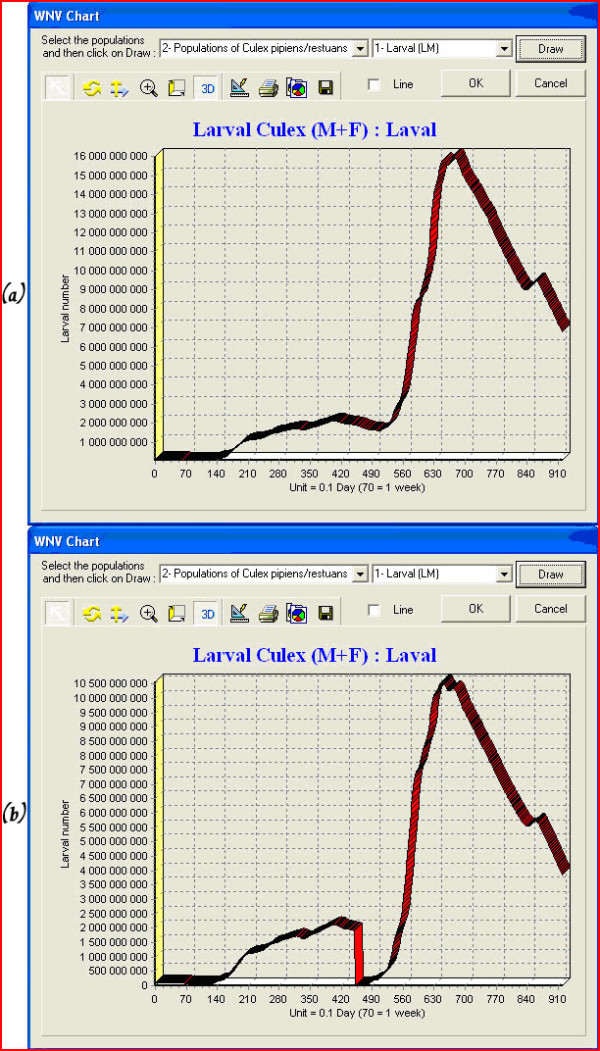
The dynamics of the larval populations before (a) and after (b) the flushing of sumps in Laval Municipality on August 15^th ^(hypothetical scenario defined by user).

In our system, multi-agent geosimulation is at the heart of a decision support tool. Hence, our approach is somewhat different from more traditional simulations used for prediction purposes [19]. The WNV-MAGS System simulates the WNV epidemics and enables a user to specify scenarios in order to explore various situations including climate change and different intervention strategies. The user may choose one among four different scenarios which influence the dynamics of the *Culex *population (Figure 6). The first scenario is the default scenario which can be set in order to use average conditions of temperature and precipitations (using in this case the Canadian Climate Normals [20]). In order to estimate the number of mosquitoes located in each municipality, we computed the number of sumps that are along the municipality's roads (see section 4.3). Sumps offer ideal locations for the maturation of larvae and the emergence of adult mosquitoes. They are also the main targets of larvicide spraying. But abundant rains may flush sumps, killing a large proportion of larvae. In a second type of scenario, the user can choose a date during which abundant rains may flush sumps in some municipalities (Figure 7). In the same way, the third scenario is used to simulate the use of larvicides in a certain area (municipality). The last scenario is a combination of the second and third scenarios. Hence, it is possible to choose a date for the flushing of sumps and another date for the application of larvicides. Most larvae are supposed to die after the flushing of a sump, although the dynamics of the larval populations starts all over again since there are always *Culex *adults in the vicinity of the sump that will spawn new eggs. Moreover, the WNV-MAGS System offers a variety of functionalities to the user in order to modify the parameters of the mathematical model, to visualize the progress of the infection in and around the crows' roosts, to extract data from the simulation and to generate graphs showing the evolution of the involved populations.

### Calibration of the system

The qualitative results of the model which represent the distribution of the populations were satisfactory. Indeed, the resulting curves reflect the biological behaviours of the studied species according to the opinion of the consulted domain experts (from GDG and UQTR). However, the quantitative data needed to be calibrated in order to be used in real-life situations. In fact, we calibrated the model by comparing simulation results and field observations (ISPHM-WNV data [4]). We evaluated the ratio between the real populations of mosquitoes and the samples of mosquitoes captured in traps (absolute densities) as well between crows and the collected dead crows.

Regarding the populations of *Culex*, we used Reisen's work [21,22] to estimate the mosquito density ratio. A captured mosquito was considered to represent a population of 300 *Culex *over one km^2^. Since we did not have data for all regions, we only calibrated simulation results for some key municipalities where human infections had occurred. It appeared thereafter that there was a significant difference between the data generated by the model and those obtained from the field. Hence, we tuned up the initial settings of the simulation (e.g. the initial percentage of infected *Culex *or infected crows, distance between sumps, emerged *Culex *per sump, percentage of sumps containing larvae, etc) as well as some parameters of the mathematical model (e.g. mosquitoes biting rate of crows per capita, WNV transmission probabilities from *Culex *to crows of from crows to *Culex*, etc). These changes have helped us to quantitatively calibrate the model for the processed municipalities.

**Figure 8:**
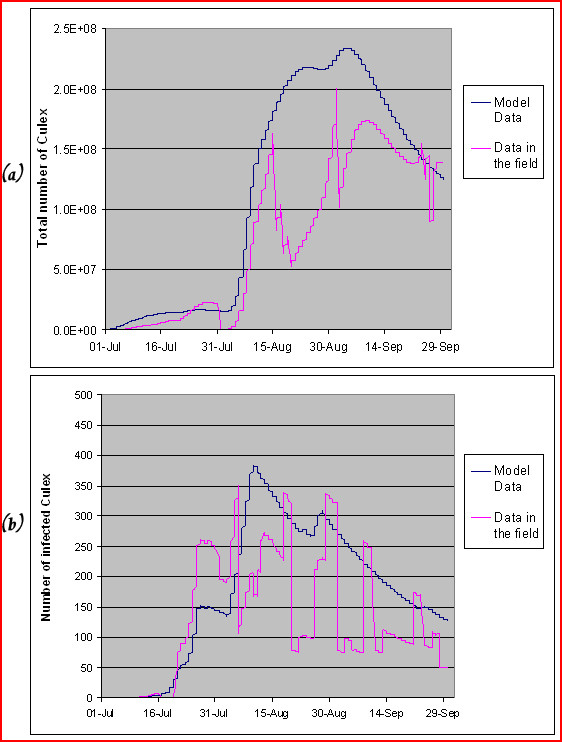
Model calibration using the average total mosquitoes captured in traps (a) and those among them which are infected with the WNV (b) during the considered simulation period (1 July – 1 October) for Laval Municipality (2003 to 2006).

Figure 8a presents the evolution of the total number of mosquitoes for the municipality of Laval between July 1^st ^and October 1^st^. The smooth blue curve represents the data generated by the simulation while the rugged red curve represents averages of real data over four years (2003 to 2006). We had to consider these averages because we do not have sufficient data from the field (trap measurements are sparse and not carried out regularly in Quebec municipalities). Moreover, these data averages enabled us to adjust our initial data in the simulation (mainly the initial number of mosquitoes) as it can be observed in Figure 8a. The simulation curve and the real data curve fit nicely between July 1^st ^and August 15^th^. The two big drops that are observed in the real data curve are difficult to explain at this point since we consider the average measures over four years. This may be the result of systematic larvicide applications in July and August (3 applications in some municipalities during a WNV season), but we have no sufficient data to confirm this conjecture. In figure 8b, we also observe a similarity between the curves representing the infected mosquitoes. Again, the rugged red curve represents averages of real data over 2003–2006. All drops in the curve result from lack of sufficient field data.

Regarding the populations of crows, we used the results presented by David and colleagues [23] in order to determine whether the numbers of dead birds sighted and tested for WNV are representative of the true bird mortality. We also used the index trend obtained from the *ÉPOQ *database [24] and from the North American Breeding Bird Survey [25] to adjust the population of crows as well as the population of generic birds. Moreover, changes in the population of crows have been calibrated using field data collection of dead birds and their analyses in the laboratory, as it was done for the population of *Culex*.

**Figure 9:**
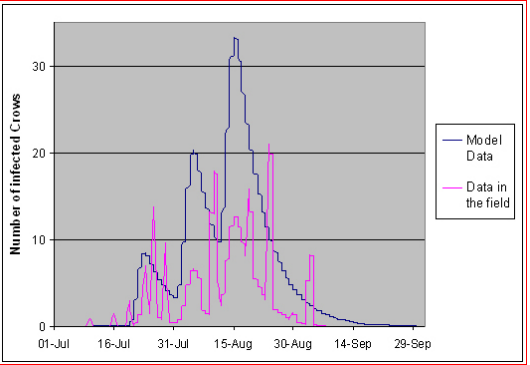
Model calibration using the average collected dead birds during the considered period of the simulation (1 July – 1 October) for Laval Municipality (2003 to 2006).

Figure 9 represents a comparison of the simulated data (smooth blue curve) and real data (red rugged curve) for the collected dead crows. The general shapes of the curves are similar. This is encouraging since data available for dead birds are even sparser than for mosquitoes.

**Figure 10:**
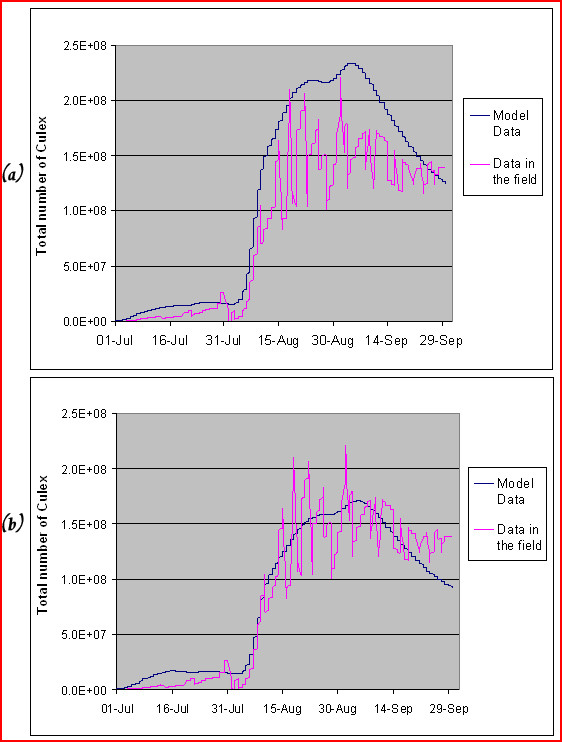
Model calibration using the total mosquitoes captured in traps during the simulation period (1 July – 1 October) for Laval Municipality in 2003: (a) without larvicide application, (b) with three larvicide applications (18 June, 17 July, and 13 August).

Then, we looked at real data for Laval Municipality for 2003, the year for which we have the most complete data set. We created the temperature scenario for 2003 and launched the simulation. Figure 10a presents the difference between the simulated data (blue smooth curve) and the real data. In order to explain this difference, we checked with the SOPFIM Company if larvicides had been sprayed in Laval Municipality in 2003. It was indeed the case, with interventions on June 18, July 17, and August 13. We created a new scenario using these three dates for larvicide spraying and we got the curve displayed in Figure 10b. The curve of simulated data now approximates the real data fairly well (the rugged curve of real data being again explained by missing data). This is an encouraging result showing that the parameters adjusted for calibration provide reasonable results.

In order to improve precision and validate our models and the simulation parameters, we will carry out the simulation on a different data set. We are currently collecting data (for mosquitoes and crows) for the city of Ottawa. We expect to get a more complete data set, since measurements have been more frequent and regular in the Ottawa region (Canada) over the past 6 years. This work is in progress.

## Conclusion and future work

In this paper, we presented a system that simulates the interactions of the populations of mosquitoes and birds which are involved in the propagation and transmission of the WNV. Moreover, we used a multi-agent geosimulation approach which takes into account the influence of the geographic characteristics of the various regions, thanks to the use of GIS data. For example, we determined the geo-referenced co-ordinates of crows' roosts in order to locate them on the map and we were able to develop rules which control the expansion/contraction of roosts over space and time. We also pre-processed climate data in a GIS in order to feed it to the simulation. We also used the geographic characteristics and the location of favourable habitats for mosquitoes in order to represent the populations of *Culex *using an intelligent density map. Consequently, we were able to implement the interactions between the mosquitoes' and birds' populations which can cause an outbreak of the virus and epidemic propagation of the disease. Even if other works [26,27] also used GIS data to simulate the spread of the WNV, they did not offer a decision support system as we do. In contrast, our system enables users to simulate the propagation of WNV under various short-term climate scenarios and allows for local parameterization. This approach may be useful for practical decision-making. For instance, it has been shown [28] that the number of degree-days below -5°C in the winter and the number of degree-days greater than 25°C in the summer may contribute to a highly epidemic emergence of the virus during the summer under specific climatic conditions. Consequently, our system may be used to predict such an epidemic if we simulate the propagation of the WNV using a scenario in which seasonal forecasts of climatic data are favourable for the emergence of the virus [29]. By assessing the simulation results and comparing the outcomes of different intervention scenarios, the users of the WNV-MAGS System can make more informed decisions about the actions to be taken such as the application of larvicides or the stepping up of personal protection measures.

An important limit of this kind of approach is the lack of field data. As we have already shown in this paper, a good calibration and validation of the models depends on the availability of a large variety of data sets (related to mosquitoes and to different species of birds). There is also a difficulty in estimating the parameters needed in the mathematical model, which would require in some cases that additional field studies be carried out by entomologists and ornithologists. In addition, the potential effects of changing resistance and immunity in wild birds remain unknown and need to be studied by domain experts. Obviously, they have not been included in our models yet.

If we were able to collect sufficient data about the WNV spread in different regions during the past years, we could develop scenarios and simulations whose results could be compared to recorded field data. Consequently, we would be able to further validate the system and adjust the various parameters that are used for the simulations, taking into account the specificities of the considered regions and species.

Nonetheless, the system can already be used to compare different scenarios involving variations of the climatic data in relation to the potential spread of WNV in particular regions. As we have shown, the system can also be used to estimate the influence of human intervention based on larvicide application. However, since it still remains difficult to get accurate weather forecasts over long periods (several months), public health authorities will have to take into account this inherent limitation of meteorological science when developing intervention plans using such a tool.

Our MAGS approach and tool can be used not only to simulate the propagation of the WNV, but they can also be adapted to various other vector-borne diseases. We are currently working on the simulation of Lyme disease in Quebec. Moreover, the tool and approach can be extended to take into account the specificities of other similar diseases (e.g. SARS) in other geographic areas.

## Methods

In order to develop the WNW-MAGS System, we applied an 'Agile' [30] analysis and design method which favours the collaboration with domain specialists and users, as well as quick adaptations of the software under development. We also applied classical knowledge engineering techniques [31] in order to acquire domain knowledge from the specialized literature and from domain experts (entomologists and ornithologists) after many work sessions. We then went through an exploration phase of the field by gathering the maximum useful information in order to understand all the phenomena which are related to the spread of WNV. We present in this section the conceptual architecture which is used as a basis of the simulation system. We also present the mathematical model which was chosen and adapted in order to compute the dynamics of the populations involved in the transmission of the WNV. We also present a subset of the relevant data which is used to feed the system. Finally, we present the implantation of the system.

### Conceptual architecture

**Figure 11:**
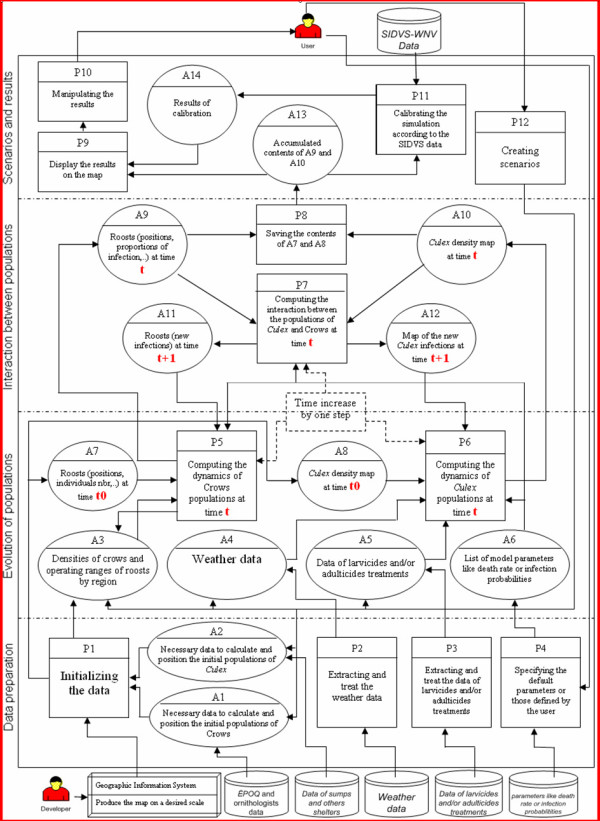
**Conceptual architecture of the system (based on*****EPAS*****method**[32]).

Based on the requirement specifications and using the conceptual model of the phenomena (see Figure 2 in section 2.2), we designed a conceptual architecture of the WNV-MAGS system which includes all the needed system components and their relationships [32]. While constructing this architecture, we identified all the processes to be developed (represented as rectangles numbered as *Pi *in Figure 11) and all the data stores (represented as ovals numbered as *Ai *in Figure 11) that gather data and feed the system processes. Indeed, most of the necessary data are obtained from external databases (*EPOQ *[24], Weather data, etc.) and GIS. They are represented as 'cylinders' at the bottom of Figure 11.

**Figure 12:**
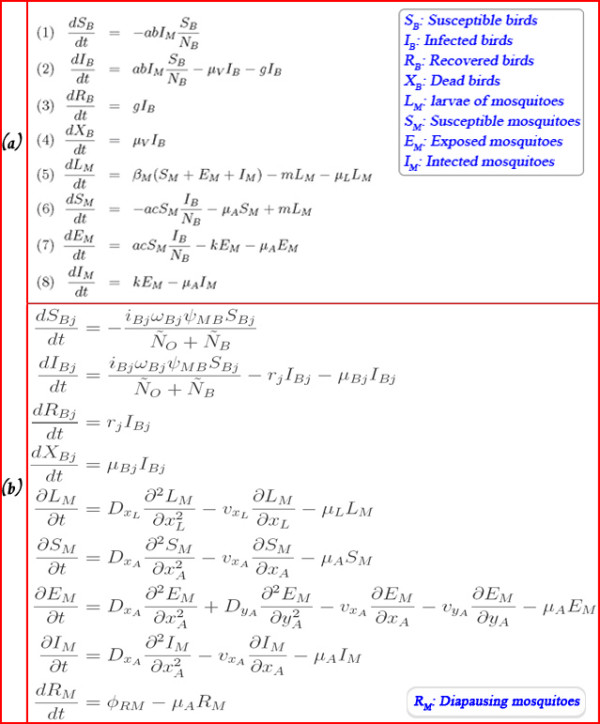
**(a) Differential equations of Wonham's mathematical model**[16]**, and (b) the new equations of the proposed model**[18].

The architecture is divided into four parts. The first part (processes P1 to P4) deals with data preparation, including the extraction of data from all the required databases. The second part (processes P5 and P6) computes the evolution of populations using the mathematical model presented in Section 4.2. The third part (processes P7 and P8) deals with the interactions between the sub-populations of crows and the sub-populations of *Culex*. It reflects the interactions between the agents' roosts and the intelligent density map. The last part (processes P9 to P12) is responsible for the management of scenarios, as well as for the display, analyses and calibration of the results. We used different databases (as presented in Section 4.3) in order to initialize the populations of *Culex *and crows at time *t*_*0 *_(beginning of the simulation). We used these populations to compute the dynamics of crows, the dynamics of *Culex *and the interactions between the two populations while increasing the time by one step, at time *t*. Then, we get the new populations at time *t+1 *(taking into account the state changes of the sub-populations of mosquitoes and crows reflecting the infection spread and deaths) and the system triggers again the same processes to simulate the joint evolution of the two populations. The simulation results can then be displayed on the map. Domain specialists can also calibrate the simulation using the WNV surveillance data (that we have pre-processed). The user can manipulate the results and create various scenarios. Then, the simulation results can be assessed and compared.

### Mathematical model

We need to compute the evolution of the populations of *Culex *and the populations of crows in order to simulate their interactions using the geosimulation system. To this end, we selected the model proposed by Wonham and colleagues [16] to compute the dynamics of the two populations. This model is based on 8 differential equations (Figure 12a) which can compute over time the evolution of the different categories of individuals (called 'compartments'): *susceptible*,*infected*, *recovered *and *dead *birds, the *larvae *of mosquitoes and the *susceptible*, *exposed *and *infected *adult mosquitoes. We proposed some modifications in order to correct some discrepancies that we found in the model. We also included climate effects in the model using the work of Madder and colleagues [33] as a starting point. This was a difficult task because the model was no longer in equilibrium and this required several modifications to the differential equations [18]. Figure 12b presents an overview of the new equations of the proposed model. We notice that the birds equations (*dS*_*BJ*_/*dt*, *dI*_*BJ*_/*dt*, *dR*_*BJ*_/*dt*, *dX*_*BJ*_/*dt*) have an index *j *which represents a different bird species that we want to include in the simulation. Climate effects are computed using another set of equations that is not presented in this paper. These equations modify certain parameters in the differential equations of Figure 12b. For example *v*_*xL *_in the *dL*_*M*_/*dt *equation represents the rate of progress of larvae toward the state of nymphae, this rate depends on temperature conditions defined in a different set of equations. The adjusted model gives satisfactory results in terms of quality (e.g. distribution of the mosquitoes' generations). Indeed, the pace of the established curves reflects the biological behaviours of the studied species if we refer to the specialized literature. However, the quantitative results (e.g. the number of larvae, eggs, emerged *Culex*, dead crows, etc.) had not been conclusive with the first results of the simulation. We corrected this problem with the calibration of the system as presented in Section 2.4.

### Preparation of data

We already mentioned that our approach takes advantage of GIS data in order to properly locate the agents' roost in space. Indeed, we used the Geomedia GIS software in order to handle the geo-referenced data of the DMTI Spatial (*CanMap Streetfiles*) and the digital maps of *INSPQ*. Using this data, we created the bitmap from which the MAGS platform generates the simulation environment. This bitmap contains polygons representing a list of 945 municipalities (out of a total of 1476 for the whole province of Quebec) being part of the ecumene (inhabited part of the studied region) of the geographical area of interest. Moreover, this bitmap is also used by the intelligent density map presented earlier (see Figure 3 in section 2.3).

In addition, we had to pre-process all the data needed to feed the system (see processes *P1 *to *P4 *of the conceptual architecture in Figure 11, section 4.1). We estimated the initial populations of *Culex *and crows at the beginning of the simulation. For the population of crows, we used the *SAS *statistical software and the *MapInfo *GIS to compute a specific density of birds per region (number of individuals by square kilometer). This done by estimating an average of the sighting mentions provided by professional or amateur ornithologists (from 1997 to 2005 and located inside the ecumene) using the *ÉPOQ *database [24] (Table 2). We also processed the data relative to crows' roosts (obtained through an email survey involving expert birders or extracted from the *ÉPOQ *database [24]). This data included the co-ordinates (latitude/longitude) as well as an approximate number of individuals for each mentioned roost. We computed an average of the number of individuals in the case of several records of the same roost (same latitude and same longitude). This data is used to characterise the roost agents (Table 3). Moreover, we studied the literature and discussed with domain experts in order to collect relevant information about crows' behaviours. We used this information in order to specify some behavioural rules for the roost agent such as the way that particles spread around the roost to simulate the birds' daily search for food.

**Table 2:** Results of SAS analysis to compute an average density of crows per area by considering the mentions extracted from the *ÉPOQ *database (1997–2005) [24] and which are located inside the ecumene.

**Code _RSS**	**Name_RSS**	**Sup.** ** ecumene**	**Nb** ** Years**	**Average**	**Min.**	**Max.**	**Sum** ** _RSS**	**Density ** **(Nb/km^2^)**
4	Mauricie-Centre-du-Québec	11760	8	415	63	814	3320	0,035289116
15	Laurentides	10990	8	2367,125	23	4801	18937	0,21538899
5	Estrie	10430	8	2478,375	573	4718	19827	0,237619847
7	Outaouais	11950	7	5205,142857	364	7727	36436	0,435576808
12	Chaudière-Appalaches	15160	8	6616	2	23655	52928	0,436411609
14	Lanaudière	7060	8	3503,875	324	6913	28031	0,496299575
3	Québec	6896	8	9496,75	136	12794	75974	1,377138921
16	Montérégie	11270	8	27858,875	11323	40836	222871	2,471949867
13	Laval	246,6	8	652,75	193	1149	5222	2,646999189
6	Montréal-Centre	500,9	8	3088,375	1411	5645	24707	6,165651827

**Table 3:** Mentions of roosts reported by ornithologists or extracted from the *ÉPOQ *database (1997–2005) [24].

**Nb.**	**Lat.**	**Long.**	**Place of the roost**	**Number of ** **mentions**	**Average number** ** of crows**
1	48,43	-71,07	Bonsai (Chicoutimi)	1	40
2	47,07	-70,80	Cap Tourmente	1	500
3	45,45	-73,32	Carignan	1	100
4	46,72	-71,27	Charny	1	450
5	45,58	-71,62	Dudswell	1	70
6	45,00	-74,05	Dundee	1	200
7	46,73	-75,47	Ferme-Neuve	1	450
8	45,24	-72,44	Granby_1	1	8000
9	45,39	-72,75	Granby_2	2	20450
10	46,02	-73,45	Joliette	1	200
11	48,03	-70,87	La Baie	1	1500
12	48,53	-71,38	Lac Duclos	1	150
13	45,08	-73,37	Lacolle	1	200
14	46,80	-71,18	Lévis	2	161
15	46,54	-75,45	Mont-Laurier	1	300
16	46,55	-75,05	Mont-Laurier, ruisseau Villemaire	3	222
17	45,48	-73,67	Montréal	1	3000
18	47,58	-68,80	Notre-Dame-du-Lac	6	780
19	46,75	-71,13	Pintendre	1	185
20	48,43	-68,55	Rimouski	4	71
21	48,48	-68,43	Saint-Anaclet	1	563
22	45,30	-73,23	Saint-Athanase, rivière Richelieu	2	5000
23	48,05	-68,25	Saint-Donat (Rimouski)	2	575
24	45,62	-72,95	Saint-Hyacinthe_1	1	1000
25	45,63	-72,98	Saint-Hyacinthe_2	1	300
26	45,32	-73,27	Saint-Jean-sur-Richelieu	5	3540
27	48,47	-78,05	Saint-Marc-de-Figuery	1	1300
28	45,25	-71,54	Sherbrooke	1	200
29	45,52	-71,81	Stoke	1	150
30	46,02	-73,15	Tracy	21	825
31	46,05	-71,91	Victoriaville	1	500
32	47,55	-68,65	Ville-Dégelis	1	400

Considering the *Culex *population, we computed the number of individuals of the initial population, estimating the number of adults that emerge from the larvae laid down in sumps (which we supposed to be the main reservoirs of mosquitoes in urban and sub-urban areas). To this end, we developed a Visual Basic application in order to query the geo-referenced databases in Geomedia and to compute the total length of roads for each municipality of interest. We then computed the number of sumps in each municipality by using the total length of roads (Table 4). We assumed that there is an average of one sump for each 30 linear meters of road. This average distance conforms to the standards used by the *Ministère des transports du Québec *(MTQ) when they install sumps. However, the user of the WNV-MAGS system can modify this value, since it can vary in relation to the considered regions such as urban and rural areas. We also assumed that there are only 20% of sumps containing larvae. This default value can also be modified by the user as well as the average number of *Culex *adults which emerge from a sump at the beginning of a simulation. These numbers were obtained by consulting data provided by the *SOPFIM *Company [34] in relation to the monitoring of larval and adult *Culex *in certain Quebec regions during the summer of 2005.

**Table 4:** Example of the results obtained in order to calculate the length of the roads of the municipalities using the data of the DMTI Spatial (CanMap Streetfiles).

**Municipality of Sainte-Foy**			
**File**	**Description**	**Number of road's pieces**	**Total length of the roads** ** (m)**

QChwy	*Highways*	100	20406,61
QChrd	*Major Roads and Highways*	435	58876,30
QCrds	*Roads*	2921	441635,34

			
		Total :	**520918,25**
		Nb. sumps if there is 1 sump/30 m	**17364**

Furthermore, we used a DLL which enables us to integrate the climate data into the system. This DLL represents one of the functionalities of the *BIOSIM *software [35]. We used it to interpolate values for temperature and precipitations at certain precise locations on the territory, taking into account the data of the four neighbouring weather stations and the elevation data. This computation can be done using either real data or the *Canadian Climate Normals *[20] which are produced over several years.

### Implementation of the system

**Figure 13:**
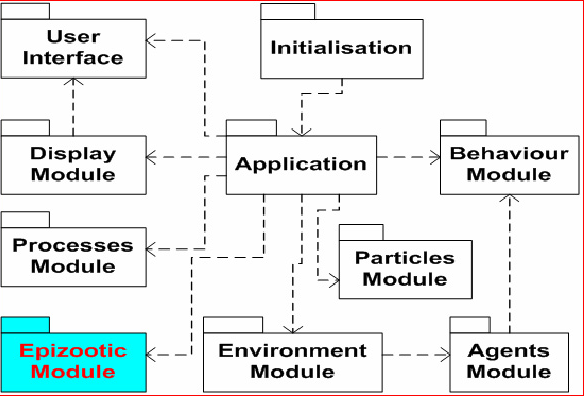
Various components of the technical system architecture (the epizootic module was added to the pre-existing MAGS components).

After the data preparation, we implemented the system using the MAGS platform which is developed in C++. This simulator includes several modules performing various tasks. It contains a controller to manage the threads of application (Processes Module), a user Interface and a module managing the simulation environment (Environment Module). In addition to these modules, MAGS contains a module in charge of data display, modules to specify agents' populations and agents' behaviours, and a module simulating particle systems. In order to simulate the propagation of the WNV, we added a module which simulates an epizooty (Epizootic Manager) which has been developed in a generic way in order to be easily extended to simulate epizooties different from WNV (Figure 13).

## Abbreviations

CHUQ: Centre hospitalier universitaire de Québec; DLL: Dynamically-linked library; ÉPOQ: Étude des populations d'oiseaux du Québec; GDG: GDG Environnement; Contrôle biologique des insectes piqueurs; GIS: Geographic information system; INSPQ: Institut national de santé publique du Québec; ISPHM-WNV: Integrated system for public health monitoring of West Nile Virus; MAGS: Multi-agent geosimulation; MSSS: Ministère de la santé et des services sociaux; MTQ: Ministère des transports du Québec; RSS: Régions socio-sanitaires; SOPFIM: Société de protection des forêts contre les insectes et maladies; UQTR: Université du Québec à trois-rivières; WNV: West Nile virus.

## Competing interests

The authors declare that they have no competing interests.

## Authors' contributions

MB was in charge of the development and implementation of the system, and was responsible for the writing of the paper, BM was the project leader, involved in the development of the system and in the decision-making process. He participated in the writing and revision of the paper, PG was our public health expert and involved in the development of the system and in the decision-making process. He participated in the writing and revision of the paper. All authors read and approved the final manuscript.
